# Mapping Competence in Gastrointestinal Endoscopy Nursing Practice: An Item Response Theory Analysis of Perceived Skill Acquisition and Maintenance in Italy

**DOI:** 10.3390/healthcare14020203

**Published:** 2026-01-13

**Authors:** Mattia Bozzetti, Gennaro Pascale, Ilaria Marcomini, Alessio Lo Cascio, Fabio Grilli, Caterina Sclapari, Grazia Multari, Nicoletta Orgiana, Mirko Gaggiotti, Giorgio Iori, Luciana Nicola Giordano, Stefano Mancin, Fabio Petrelli, Giovanni Cangelosi, Loris Riccardo Lopetuso, Daniele Napolitano

**Affiliations:** 1 Direction of Health Professions, ASST Cremona, 26100 Cremona, Italy; mattia.bozzetti@asst-cremona.it; 2 AORN Sant’Anna e San Sebastiano, 26100 Caserta, Italy; gennaro.pascale@aorncaserta.it; 3 Center for Nursing Research and Innovation, Faculty of Medicine and Surgery, Vita–Salute San Raffaele University, 20132 Milan, Italy; marcomini.ilaria@unisr.it; 4 Direction of Health Professions, La Maddalena Cancer Center, 90146 Palermo, Italy; locascio.alessio@lamaddalenanet.it; 5 Endoscopia Digestiva Chirurgica–Fondazione Policlinico Gemelli IRCCS, 00168 Rome, Italy; fabio.grilli@policlinicogemelli.it; 6 UOC Gastroenterologia ed Endoscopia Digestiva, G.O.M Bianchi Melacrino Morelli, 89124 Reggio Calabria, Italy; caterina.sclapari@gmail.com; 7 SOD di Chirurgia Endoscopica, Dipartimento Assistenziale di Area Medica-Presidio ‘Pugliese’, Azienda Ospedaliera Universitaria ‘Renato Dulbecco’ di Catanzaro, 88100 Catanzaro, Italy; graziamultari3@gmail.com; 8 CUT, Fondazione Policlinico Gemelli IRCCS, 00168 Rome, Italy; nicoletta.orgiana@policlinicogemelli.it; 9 Clinica di Gastroenterologia ed Epatologia, Ospedale Santa Maria Della Misericordia, Azienda Ospedaliera di Perugia, 06129 Perugia, Italy; 10 Gastroenterologia ed Endoscopia Digestiva di Reggio Emilia, Azienda USL di Reggio Emilia IRCCS Ospedale S. Maria Nuova, 42123 Reggio Emilia, Italy; iori.giorgio.re@gmail.com; 11 CEMAD, Fondazione Policlinico Gemelli IRCCS, 00168 Rome, Italy; luciananicola.giordano@policlinicogemelli.it (L.N.G.); lopetusoloris@gmail.com (L.R.L.); daniele.napolitano@policlinicogemelli.it (D.N.); 12 IRCCS Humanitas Research Hospital, 20089 Milan, Italy; stefano.mancin@humanitas.it; 13 School of Pharmacy, Experimental Medicine and “Stefani Scuri” Public Health Department, University of Camerino, 62032 Macerata, Italy; 14 Department of Life Science, Health, and Health Professions, Link Campus University, 00165 Rome, Italy

**Keywords:** nursing, competence, clinical and assistive skill, cross-sectional survey, Italy

## Abstract

**Highlights:**

Advanced gastrointestinal endoscopy requires specific nursing competences to ensure procedural safety, efficiency, and patient-centered care. International frameworks define core skills and assessment methods, but Italy lacks a standardized, nationally recognized competency model, leading to variability in training and practice.

**What are the main findings?**

- This study quantifies perceived procedural repetition thresholds for both competence acquisition and maintenance across 30 endoscopic procedures. It demonstrates that complex or infrequently performed techniques require higher perceived thresholds and that more experienced nurses set stricter internal standards. The findings provide a validated, procedure-specific competence map for Italian endoscopy nursing.

**What are the implications of the main findings?**

- The results support the development of national competence standards, modular and adaptive training programs, and structured mentorship for gastrointestinal endoscopy nurses in Italy. Adoption of such a model could harmonize practice, enhance skill development, and improve patient outcomes.

**Abstract:**

**Objective**. The aim of this study was to define a structured competence model for nurses working in gastrointestinal endoscopy in Italy and to assess nurses’ perceptions of the number of procedural repetitions required to acquire and maintain competence across different endoscopic procedures. **Methods**. A cross-sectional online survey targeted registered nurses working in Italian gastrointestinal endoscopy units. The questionnaire, developed from guidelines and expert consensus, covered demographics, organizational context, and perceived repetition thresholds for 30 procedures. Partial Credit Models (PCMs) estimated acquisition and maintenance thresholds; Differential Item Functioning (DIF) tested differences by self-reported experience level. **Results**. A total of 332 nurses participated (68.4% female; mean age 47.1 years; mean endoscopy experience 10.1 years). For competence acquisition, most procedures were placed in the 11–30 or 31–50 repetition range, with higher values for complex techniques. Competence maintenance generally required fewer repetitions, but thresholds varied by procedure. Advanced or infrequently performed techniques were perceived as more demanding. More experienced nurses reported higher thresholds, reflecting stricter internal standards. **Conclusions**. Acquisition and maintenance of gastrointestinal endoscopy competences differ in intensity and frequency requirements, supporting the need for tailored, modular training pathways. Findings highlight the importance of national competence standards, adaptive learning technologies, and structured mentorship to enhance skill development, reduce variability, and promote consistent, high-quality patient care across Italy.

## 1. Introduction

In recent years, healthcare systems have undergone significant transformation, driven by technological innovation, increasing clinical complexity, and a growing emphasis on patient involvement [1,2]. Within this evolving context, the nursing role has shifted from a task-oriented to an autonomous, specialized professional capable of operating in technologically advanced and relationally demanding settings [3]. This evolution is particularly evident in gastrointestinal endoscopy, where nurses’ technical, managerial, and communicative competences have become increasingly central. The growing complexity and volume of procedures, such as endoscopic submucosal dissection (ESD), cholangioscopy, and thermal ablation, have highlighted the need for advanced skills [4,5]. Targeted training in instrument handling not only reduces equipment-related costs but also improves service quality [6,7]. International societies such as European Society of Gastroenterology and Endoscopy Nurses and Associates (ESGENA) and European Society of Gastrointestinal Endoscopy (ESGE) emphasize the importance of structured training, as outlined in their joint guidelines, which define core endoscopic procedures, the required technical and cognitive skills, and the use of evaluation tools such as Key Performance Indicators (KPIs), Direct Observation of Procedural Skills (DOPS), and logbooks [8,9,10].

Recently, an Italian consensus has defined a three-tier competence model (i.e., trainee, competent, and advanced) that also incorporates non-technical skills [11]. Beyond technical expertise, nurses support pain and anxiety management, improving patient experience and procedural outcomes. Their presence has been associated with increased detection rates and reduced complication rates in colonoscopy, as well as shorter procedure times and better outcomes overall [12,13,14]. Moreover, the presence of a dedicated nurse during colonoscopy has been associated with a significant increase in polyp and adenoma detection rates, thereby enhancing the clinical effectiveness of the procedure [15,16]. In terms of clinical outcomes, several studies report that nursing experience in endoscopy correlates with reduced complication rates, shorter procedure times, and improved outcomes such as cecal intubation rates [17,18]. Despite growing evidence, Italy lacks a standardized, institutionally recognized model defining nurses’ competences in gastrointestinal endoscopy. The role is often described locally, leading to variability in training, responsibilities, and assessment criteria [11], which can affect care quality and professional development. Current Italian competency models for gastrointestinal endoscopy nursing remain primarily descriptive and lack empirically derived procedural thresholds to guide training intensity, progression, and skill maintenance. A recent scoping review highlighted substantial variability in the duration and content of endoscopy nursing training programs across Europe, including Italy. It emphasized the absence of standardized, evidence-based benchmarks for procedural repetition and skill acquisition [19]. Although international societies recommend the use of validated assessment tools and performance indicators in endoscopy training, these recommendations primarily pertain to physicians, and no empirical data currently define the number of procedural repetitions required for nurses to acquire or maintain competence in specific endoscopic techniques [20,21]. Moreover, existing competence assessment tools for endoscopy nurses focus on multidimensional skill evaluation but do not address procedural volume requirements, further limiting the development of structured, evidence-informed educational pathways [22,23,24].

Therefore, this study aimed to psychometrically examine nurses’ perceptions of procedural repetition thresholds required for competence acquisition and maintenance in gastrointestinal endoscopy and to explore differences by self-reported experience within the Italian context.

## 2. Materials and Methods

### 2.1. Study Design

This study employed a cross-sectional, survey-based design, using an online questionnaire to collect data on the training needs and professional competences of nurses working in gastrointestinal endoscopy units across Italy. For transparency and scientific validity, the manuscript was prepared according to Strengthening the reporting of observational studies in epidemiology (STROBE) [25], and a checklist was inserted in Supplementary Materials (Table S1).

### 2.2. Setting

The survey was conducted between September 2024 and March 2025 across multiple gastrointestinal endoscopy units located in different regions of Italy. The participating centers included a diverse mix of public hospitals, university-affiliated medical centers, research institutions, and private healthcare providers. These settings varied in terms of procedural volume, clinical complexity, and technological resources, providing a representative overview of the national landscape of endoscopic nursing practice.

### 2.3. Participants

Eligible participants were registered nurses currently employed in gastrointestinal endoscopy settings, regardless of their specific contractual status, years of experience, or educational background. A snowball sampling approach was adopted to facilitate recruitment, in which nurse managers were invited to share the survey link with colleagues in their settings. No exclusion criteria based on age, gender, geographic area, or professional title were applied to capture a broad range of professional perspectives and practice environments. As the survey was disseminated using a snowball sampling strategy, a formal response rate could not be calculated. No personally identifiable information or IP addresses were collected. Data collected through Google Forms were exported into a spreadsheet and screened before analysis. Responses were checked for completeness, and questionnaires with substantial missing data—particularly in sections related to competence acquisition or maintenance—were excluded. To reduce the risk of duplicate entries, submissions were reviewed for identical response patterns across key variables (e.g., professional experience, procedural estimates, and completion timestamps). Only fully completed questionnaires with internally coherent response patterns were retained for analysis. Isolated missing values were handled using listwise deletion, as appropriate for Rasch-based modelling.

### 2.4. Study Variables

An online questionnaire (via Google Forms) was administered to explore the training needs and professional competencies of nurses working in gastrointestinal endoscopy units across Italy. The instrument was developed by a panel of clinical nursing experts, based on a review of international guidelines, existing competency frameworks, and preliminary discussions with professionals in the field [21]. Before dissemination, the questionnaire was reviewed by the expert panel to assess face validity, clarity, and content relevance. Minor refinements were made to improve wording and comprehensibility. No formal pilot testing or psychometric validation was conducted before distribution, as the primary aim of the study was exploratory. The questionnaire included both closed- and open-ended questions and was structured into six thematic sections, as detailed below.

### 2.5. Sociodemographic and Professional Characteristics

The questionnaire collected basic sociodemographic and professional information (e.g., region of current employment, age, gender, total years of professional nursing experience, years specifically spent working in gastrointestinal endoscopy, and any experience in coordinating endoscopy units). Additionally, educational qualifications were recorded, with attention to both undergraduate and postgraduate training, including specialist academic programs in endoscopy nursing.

### 2.6. Organizational and Educational Context

Participants were asked about their participation in conferences organized by scientific societies focused on endoscopy, previous attendance at training programs related to the assessment of nursing competences, and whether theoretical instruction is provided before introducing nurses to new endoscopic procedures. The availability of standardized protocols or checklists for procedure execution, such as room setup or patient preparation, was also explored. Furthermore, respondents were asked to specify the type of institution in which they work (e.g., public, private, university-affiliated) and the level of clinical complexity in their unit. They also identified the types of endoscopic procedures routinely performed in their setting.

### 2.7. Acquisition and Maintenance of Specialized Skills

This section of the questionnaire was designed to explore nurses’ perceptions regarding the acquisition and maintenance of competence in gastrointestinal endoscopy procedures. Two parallel item sets were used to collect data: one focused on the number of procedural repetitions deemed necessary to achieve autonomous competence, and the other on the number required to maintain such competence over time. For each of the 30 endoscopic procedures listed, respondents were asked to indicate the estimated range of repetitions using a four-point ordinal scale (where: 1 = “1–10”; 2 = “11–30”; 3 = “31–50”; 4 = “>50”). The same set of procedures was repeated for both acquisition and maintenance, allowing for a direct comparison between the learning and retention phases of professional competence. All procedures included in this section were selected based on their clinical relevance, frequency in endoscopic practice, and representation across different levels of technical complexity—from basic tasks (e.g., room preparation and instrument reprocessing) to advanced interventions Endoscopic Retrograde Cholangiopancreatography (ERCP); endoscopic ultrasound (EUS); Endoscopic Full-Thickness Resection (EFTR); Endoscopic Mucosal Resection (EMR); Peroral Endoscopic Myotomy (POEM); Percutaneous Endoscopic Gastrostomy (PEG); Percutaneous Endoscopic Jejunostomy (PEJ); Fecal Microbiota Transplantation (FMT); Endoscopic Submucosal Dissection (ESD). This methodological framework was intended to capture both the variability in individual learning curves and the practical criteria nurses apply when assessing readiness for independent practice in complex procedural environments. The study intentionally focused on self-reported perceptions of competence rather than objective performance assessments. This choice was consistent with the study’s aim of mapping perceived competence thresholds and internal standards of readiness, which reflect how nurses subjectively define the acquisition and maintenance of autonomy across different endoscopic procedures. Objective competence measures (e.g., direct observation, procedural audits, or simulation-based assessments) were not included, as the study was designed to explore perceived thresholds rather than to evaluate actual clinical performance.

### 2.8. Statistical Analysis

Descriptive statistics were calculated to summarize the demographic, educational, and professional characteristics of the sample. For continuous variables, means (M) and standard deviations (SD) were reported. Categorical variables were described using absolute frequencies (*n*) and corresponding percentages (%). To model respondents’ perceptions of the number of repetitions required to acquire and maintain independent competence in gastrointestinal endoscopy procedures, two separate Partial Credit Models (PCM) were estimated [23,24]. The PCM was chosen as it accommodates polytomous ordinal data and allows for item-specific threshold estimation, making it suitable for analyzing rating-scale responses across a heterogeneous set of tasks [25]. Each model included 30 items, corresponding to specific procedures identified as clinically relevant by the expert panel. The first model estimated parameters related to competence acquisition, i.e., the number of times a procedure should be performed to achieve autonomous execution. The second model focused on the maintenance of competence, i.e., the frequency of practice required to retain skills over time. For each item, category thresholds were estimated independently, without constraining them to be equal across items [23]. The threshold whose location on the latent ability continuum most closely matched the average person’s location was identified as the item’s “optimal cut-point,” representing the modal point of transition to perceived autonomy. This procedure enabled mapping of subjective learning curves across different procedures. Item fit was evaluated using outfit mean square (MSQ) statistics, where values between 0.7 and 1.3 were considered acceptable, reflecting good model–data correspondence without excessive randomness or response consistency [26]. Items with outfit values outside this range were flagged for potential misfit. In addition, standardized residuals were examined to evaluate the internal consistency of item responses, and Q3-like residual correlations were computed to detect local item dependencies and multidimensional patterns in the response structure [27]. Differential Item Functioning (DIF) analysis was conducted to determine whether perceived competence thresholds varied systematically across groups defined by self-reported experience level [28]. Respondents were classified into two subgroups based on their global self-assessment: “Competent/Expert” and “Beginner/Uncertain”. Participants who self-identified as “beginner” or expressed uncertainty regarding their competence level were grouped as “Beginner/Uncertain”. In contrast, those who self-identified as “competent” or “expert” were grouped as “Competent/Expert”. This grouping strategy was adopted to capture meaningful differences in internal standards of competence and professional self-perception, rather than objective skill levels. Collapsing self-reported categories into two broader groups ensured adequate group size for stable DIF estimation. It was consistent with the exploratory aim of examining whether perceived competence thresholds were interpreted differently by nurses at earlier versus more advanced stages of professional development. To assess global invariance of item parameters across these groups, Andersen’s Likelihood Ratio Test was employed. In addition, item-level comparisons of category thresholds were performed using pairwise Z-tests to detect statistically significant differences in response patterns between groups. Missing responses were minimal and were handled using listwise deletion, in line with Rasch-based modeling assumptions; therefore, only complete response patterns were included in the Partial Credit Model analyses. These analyses aimed to identify items for which the underlying latent trait was interpreted or operationalized differently, potentially indicating group-related biases or recalibrations of internal standards. No a priori sample size calculation was performed, as traditional power analysis does not apply to Rasch-based models. Sample adequacy was evaluated in relation to established methodological recommendations for Partial Credit Models, which indicate that stable estimation of item parameters can be achieved with samples of approximately 200–300 respondents for polytomous items. The final sample size was therefore considered adequate to support the analyses performed. All statistical analyses were performed in R (version 4.3.3), with the TAM [29] and eRm [30] packages used for Rasch and PCM modelling.

### 2.9. Ethical Considerations

This study involved an anonymous, voluntary survey and did not collect sensitive personal data or clinical information. According to the national law “Ministero della Salute—Decreto 30 gennaio 2023: definizione dei criteri per la composizione e il funzionamento dei Comitati etici” the study did not require ethics committee approval. However, for transparency and ethical concerns, all participants received detailed information about the study objectives, procedures, data handling, and their right to withdraw at any time. Participation was entirely voluntary, and no incentives were provided. Anonymity was ensured by avoiding the collection of identifiable information, IP addresses, or institutional identifiers, and by restricting data analysis to aggregated results only. Participants were informed that they could interrupt or discontinue the survey at any time without providing justification or facing any consequences.

## 3. Results

### 3.1. Sociodemographics

The survey encompassed cohort of 332 respondents, predominantly female (68.4%), with an average age of 47.1 (SD = 9.6) years. Participants reported substantial professional experience, averaging 23.6 (SD = 11.8) years overall and 10.1 (SD = 8.8) years specifically in endoscopy. Professional characteristics are reported in Table 1.

### 3.2. Competence Acquisition

**Figure 1:**
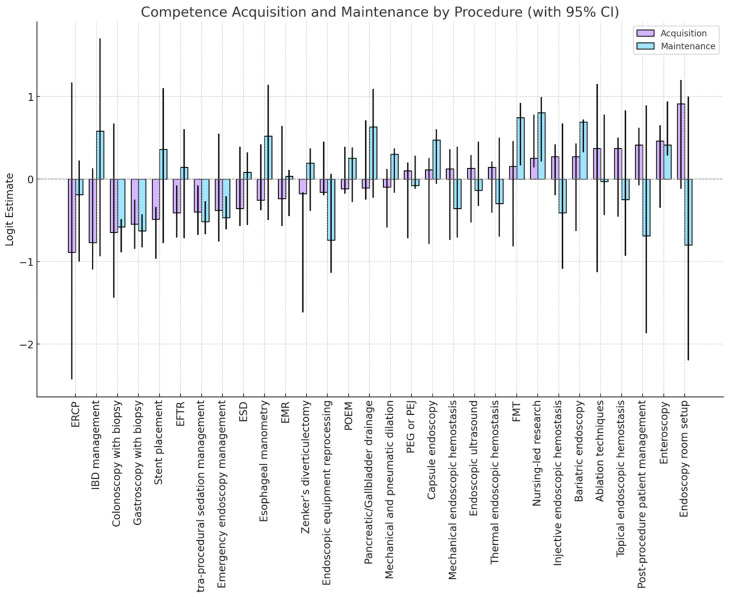
Estimated competence thresholds for gastrointestinal endoscopy procedures, separately for skill acquisition (purple) and maintenance (blue), derived from PCMs. Each bar represents the location, on a latent competence continuum (in logits), where respondents most frequently reported transitioning to perceived autonomy (“optimal cut-point”). Vertical lines indicate 95% confidence intervals. Higher logit values correspond to procedures perceived as more demanding to master. Bars further to the right denote procedures requiring more repetitions to reach or maintain independent performance. Bars with overlapping confidence intervals across acquisition and maintenance suggest similar perceived effort for both phases, whereas divergence indicates a steeper learning curve or greater retention difficulty. Procedures are presented in the original survey order to preserve contextual interpretation. Legend. ERCP: Endoscopic Retrograde Cholangiopancreatography; EFTR: Endoscopic Full-Thickness Resection; EMR: Endoscopic Mucosal Resection; POEM: Peroral Endoscopic Myotomy; PEG: Percutaneous Endoscopic Gastrostomy; PEJ: Percutaneous Endoscopic Jejunostomy; FMT: Fecal Microbiota Transplantation.

As shown in Figure 1 and Table 2, the perceived number of repetitions required to achieve independent competence varied across procedures. The optimal threshold often fell within the “11–30” or “31–50” repetition categories. Procedures such as gastroscopy with biopsy and colonoscopy with biopsy were associated with thresholds in the “11–30” range, with logit estimates of −0.55 and −0.65, respectively. Similarly, tasks such as intra-procedural sedation management and endoscopic equipment reprocessing were placed within the same range. More complex procedures, including EUS, radiofrequency ablation, enteroscopy, and Zenker’s diverticulectomy, were generally associated with thresholds in the “31–50” range. Among these, enteroscopy had the highest logit estimate (0.46), followed by radiofrequency ablation (0.37) and topical endoscopic hemostasis (0.37). The lowest logit estimate in the acquisition model was observed for ERCP (−0.89). Outfit MSQ statistics for most items fell within the acceptable range (0.7–1.3). A small number of procedures exceeded this range, such as endoscopy room setup (MSQ = 1.53) and nursing-led research (MSQ = 1.50). Outfit MSQ values above 1.3 indicate increased response variability, suggesting heterogeneous perceptions of competence thresholds across respondents rather than systematic model misfit. No items showed extreme misfit.

### 3.3. Competence Maintenance

The distribution of perceived repetition thresholds for maintaining competence was more homogeneous than for acquisition. According to Table 2, the majority of procedures were placed in the “11–30” category. This included procedures of varying complexity, such as gastroscopy with biopsy, colonoscopy with biopsy, ERCP, EUS, PEG or PEJ, and thermal endoscopic hemostasis. The logit estimates for these procedures ranged from –0.74 to −0.03. In contrast, a subset of procedures—such as Zenker’s diverticulectomy, POEM, mechanical and pneumatic dilation, and nursing-led research—were associated with thresholds in the “31–50” category. These procedures also presented the highest logit estimates in the maintenance model, including nursing-led research (0.80), FMT (0.74), and bariatric endoscopy (0.69). Most items demonstrated acceptable MSQ values for the outfit. A few procedures, including Zenker’s diverticulectomy (MSQ = 1.43) and endoscopic equipment care (MSQ = 1.42), slightly exceeded the conventional threshold but remained within interpretable limits.

### 3.4. Model Invariance and Differential Item Functioning

Model invariance was assessed to determine whether item parameters differed significantly between groups with different levels of self-assessed competence. Respondents were classified into two groups: those identifying as “Competent” or “Expert” and those selecting lower levels of competence. Andersen’s Likelihood Ratio Test (LRT) indicated significant differences in item parameters across groups for both the acquisition model (LR = 158.60, df = 99, *p* < 0.001) and the maintenance model (LR = 123.96, df = 91, *p* = 0.012), suggesting non-invariance in both cases. Pairwise Z-tests comparing threshold estimates between the two groups revealed statistically significant differences for multiple procedures, particularly in intermediate and advanced categories. These differences were observed in a broad set of items, including “post-procedure patient management”, “Gastroscopy with biopsy”, “Colonoscopy with biopsy”, “Intra-procedural sedation management”, ERCP: Stone removal, bile duct dilation, stent placement, and from PEG or PEJ through FMT. In each of these cases, the “Competent/Expert” group reported higher category thresholds compared to the less experienced group. These results were consistent across both acquisition and maintenance models, although the magnitude of differences was greater in the acquisition model.

## 4. Discussion

### 4.1. Variability of Competence Thresholds Across Endoscopic Procedures

Our study results confirm marked heterogeneity in the subjective perception of competence thresholds across various endoscopic procedures. Basic procedures are perceived as relatively accessible, whereas more complex ones, such as ESD, POEM, and EFTR, are considered significantly more demanding in terms of repetitions required to achieve autonomy. The relatively low perceived acquisition difficulty observed for ERCP, compared with other advanced endoscopic techniques, may reflect the high level of procedural standardization, structured team-based workflows, and concentrated exposure typical of ERCP practice in specialized centers, which can facilitate earlier perceived competence. Our study represents a significant step in structuring nursing competencies in gastrointestinal endoscopy within the Italian context, where institutional and standardized training models are still lacking. The PCM, a sophisticated psychometric model for ordinal data, provides a nuanced analysis of perceived competence thresholds. Beyond capturing item difficulty, the model’s Outfit Mean Square values revealed notable inconsistencies for less standardized tasks—such as independent nursing research and endoscopy room setup—highlighting a pressing need for clearer national standards. This variability is not unique to endoscopy but is a common phenomenon across many highly specialized nursing fields. Similar learning curve patterns have been reported in other high-complexity nursing specialties, including anesthesia, intensive care, and oncology nursing [31,32,33].

### 4.2. Acquisition Versus Maintenance of Competence: Educational Implications

These data suggest that educational programs must be highly tailored not only according to procedures but also considering specialty-specific characteristics, ensuring personalized and flexible training pathways that reflect learning curves unique to each clinical context. The distinction between acquisition and maintenance of competence also aligns with observations in other disciplines. This distinction has important implications for educational planning, particularly in differentiating the intensity of initial training from strategies aimed at skill retention over time [34,35]. This finding supports the introduction of frequency-based criteria for continuing education that can be tailored to procedural complexity and the need for skill reinforcement over time. Recognition that the maintenance phase requires fewer repetitions to sustain autonomy supports the adoption of targeted continuing education programs that utilize advanced simulation, distance learning, and periodic refresher sessions. Such strategies have already been successfully implemented in other high-complexity specialties such as interventional cardiology and trauma management [36].

### 4.3. Experience, Internal Standards, and Perceived Competence

The effect of internalizing higher standards by experienced professionals, also found in other nursing areas such as pain management or psychiatric care, indicates that critical awareness of one’s abilities and professional expectations increases with experience. Experienced professionals tended to set higher perceived competence thresholds, possibly due to increased self-awareness and elevated internal standards. This cognitive shift, while indicative of professional maturation, introduces subjectivity that needs to be accounted for when defining national benchmarks. Assessment systems should therefore be dynamic and stratified by experience level, avoiding a “one-size-fits-all” approach and promoting educational modalities that accompany the professional throughout their development [37].

### 4.4. Implications for Training Models and Competency Development

From a practical perspective, the empirically identified competence thresholds can inform the development of modular and adaptive training pathways structured by procedural complexity and stage of professional development. In line with ESGE and ESGENA recommendations, introductory modules can be designed for basic procedures, combining foundational cognitive training with supervised clinical exposure and simulation, while advanced techniques should be addressed through extended modules that require prior mastery of core procedures and incorporate mentored practice and progressive autonomy [38,39]. The clear distinction between competence acquisition and maintenance further supports the implementation of differentiated refresher modules, tailored to procedural frequency, scope of practice, and clinical risk, as recommended in international endoscopy safety and training guidance [38,40]. Within this framework, training intensity, duration, and content can be individualized according to prior experience and local procedural volume, supported by validated competence assessment tools and ongoing monitoring of performance indicators to guide progression and targeted reinforcement [20,22,40,41]. These findings support a shift toward more adaptive and personalized training models in endoscopy nursing [42,43]. In addition to technical competence, the study also underscores the importance of integrating relational competences—such as anxiety management, intra-procedural communication, and emotional support—into training curricula. These dimensions align with ESGENA/ESGE recommendations and reflect the holistic nature of endoscopic nursing practice [10]. Adopting a personalized vision of education and care can improve the quality of both the training experience and clinical care, promoting a more satisfying work environment for professionals and safer care for patients, in line with evolving trends in modern healthcare [44].

### 4.5. Interpretation and Applicability of Competence Thresholds

The observed variability, combined with the absence of a recognized national model, is a recurring issue in other specialties as well. The Outfit MSQ further illustrates heterogeneous perception coherence among different procedures, with routine tasks showing higher agreement and complex interventions demonstrating considerable variability. This evidence the urgent need for national standardization efforts to clarify competence requirements and reduce regional and institutional discrepancies in endoscopy nursing. For example, oncology nursing and intensive care face similar challenges, with regional variability impacting care quality and patient safety [45]. Experiences from other countries that have implemented national competency frameworks and certifications have demonstrated improvements in standardization, educational effectiveness, and professional development [46]. In this regard, adopting an Italian model grounded in evidence from this study could significantly help bridge a significant gap. The results indicate that training should be more accurately tailored to the perceived difficulty and clinical risk associated with each procedure. In other disciplines, such as critical care or advanced pharmacological therapy, training models based on differentiated competency levels (trainee, competent, advanced) supplemented by continuous formative and summative assessments have been developed [46]. Integrating the collected data into individual logbooks, combined with mentoring and supervised practice programs, represents a consolidated best practice in other nursing specialties and could be a strategic element in endoscopy as well [47]. The empirical data gathered in this study provide a valuable foundation to enhance nursing education in gastrointestinal endoscopy. Previous studies have revealed knowledge gaps among endoscopy nurses regarding IBD management [12] and have proposed a clear three-level competency framework to guide training. These findings support the need for targeted, progressive, and personalized education in gastrointestinal endoscopy nursing. Incorporating perceived competence thresholds into curricula enables the development of tailored training modules with defined learning milestones aligned with each procedure’s complexity. The adoption of digital logbooks enables nurses to record clinical experiences and self-assessments, facilitating personalized learning trajectories supported by timely feedback. Healthcare organizations should implement flexible, modular training programs that adjust in intensity and frequency according to both procedural complexity and individual experience. This flexibility supports targeted simulation exercises, focused workshops for advanced techniques, and refresher sessions on infrequently performed procedures. The adoption of online platforms for competence tracking can facilitate personalized learning trajectories by enabling detailed monitoring and feedback. Curricula structured in modular pathways allow nurses to progress according to their experience level.

Additionally, VR and AR simulation technologies provide immersive training environments, especially valuable for mastering rare or technically demanding procedures. Structured certification pathways, from trainee to competent to advanced levels, can further standardise skill development and professional recognition. Mentorship plays a critical role in facilitating skill acquisition and confidence building. Establishing formal mentoring programs pairing experienced nurses with novices promotes safe practice and continuous learning.

Additionally, integrating innovative educational technologies—such as virtual reality simulators, augmented reality tools, and interactive e-learning platforms—can enrich experiential learning opportunities [47,48]. Structured debriefings, potentially supported by video review, further enhance reflective practice and ongoing competence development. Together, these measures can significantly improve the quality, safety, and efficiency of nursing care in gastrointestinal endoscopy, while fostering professional satisfaction and career progression. Importantly, the competence thresholds identified in this study are perception-based and should not be interpreted as objective or normative indicators of clinical competence. Further validation using objective performance measures and longitudinal designs would be required before considering their use in standard-setting frameworks.

### 4.6. Study Limitations and Future Research Implications

As is common in studies based on subjective data and self-assessment, a primary limitation is the risk of selection bias. The use of a snowball sampling strategy, combined with voluntary participation, may have limited sample representativeness by favouring more motivated or professionally engaged nurses, which may influence the generalizability of the findings. Importantly, the competence thresholds identified in this study should be interpreted as perception-based benchmarks rather than objective indicators of clinical competence. They represent internalized standards of readiness and autonomy shaped by experience and context. Accordingly, their primary applicability lies in guiding educational design, mentorship intensity, and continuing training strategies, rather than serving as criteria for certification or formal competence assessment. A second limitation concerns the discrepancy between perceived and actual competence. While self-assessment provides valuable insights, it does not always align with observed performance or clinical outcomes. Evidence from other nursing fields shows that self-perception can both over- and underestimate actual competence, particularly in technical areas. Future studies should incorporate objective measures, such as direct observation, clinical audits, and outcome analysis, to validate perception-based models.

Additionally, the cross-sectional design prevents evaluation of how competences evolve over time or in response to targeted training. Longitudinal research is needed to assess the effectiveness of educational interventions and track the alignment between perceived and actual competence. Organizational and structural variability within the Italian healthcare system also represents a confounding factor, as differences in endoscopy unit configurations may affect training opportunities and practical experience. Addressing this requires a unified strategy that integrates research, education, and governance. Developing national training frameworks, supported by official guidelines and competence certification, could help reduce regional disparities and promote consistent standards of care. Parallel efforts should focus on investing in continuous education, innovative teaching methods, and educational technologies aligned with evolving clinical demands. Finally, future research should examine how educational pathways can be adapted to individual professional characteristics and procedural complexity within gastrointestinal endoscopy nursing. These demands coordinated action among educational institutions, regulatory bodies, and healthcare organizations within a systemic, multidisciplinary approach.

## 5. Conclusions

This study provides a psychometric description of perceived competence thresholds for acquisition and maintenance across gastrointestinal endoscopy nursing procedures within the Italian context. The findings highlight substantial variability in procedural complexity and professional experience, underscoring the need for structured, adaptive educational pathways specific to this clinical setting. As the identified thresholds are perception-based and derived from a cross-sectional design, they should be interpreted as exploratory reference points to support educational planning rather than as normative or generalizable standards.

## Figures and Tables

**Table 1:** Descriptive characteristics of the sample.

Variables		*n* (%)
Age (years) M (SD)	47.09 (9.57)	
Overall Experience (years) M (SD)	23.64 (11.80)	
Endoscopy Experience (years) M (SD)	10.08 (8.83)	
Coordination Experience (years) M (SD)	1.56 (4.46)	
Gender	Female	227 (68.4%)
	Male	105 (31.6%)
Region	North	93 (28.0%)
	Center	107 (32.2%)
	South & Islands	132 (39.8%)
Degree	Bachelor’s Degree	264 (79.5%)
	Master’s Degree	26 (7.8%)
	Other	42 (12.7%)
Congress Attendance	Never	36 (10.8%)
	Rarely	202 (60.8%)
	Always	94 (28.3%)
Formal Training	Yes	176 (53.0%)
	No	73 (22.0%)
	No, but would like to	82 (24.7%)
Theoretical Training	Yes	164 (49.4%)
	No	168 (50.6%)
Checklist Use	Yes	226 (68.1%)
	No	106 (31.9%)
Organization Type	Public	205 (61.7%)
	University Public	47 (14.2%)
	Private	24 (7.2%)
	Mixed/Other (Research Institute, hybrid institutions)	56 (16.9%)
Procedure Intensity	High (e.g., ERCP, EUS, bariatric)	220 (66.3%)
	Medium (e.g., polypectomies, dilations)	90 (27.1%)
	Low (e.g., screening, diagnostics)	22 (6.6%)

Legend. M: mean; SD: standard deviation; ERCP: Endoscopic Retrograde Cholangiopancreatography; EUS: endoscopic ultrasound.

**Table 2:** Optimal number of repetitions required for both acquisition and maintenance of competence.

	Acquisition			Maintenance		
Procedures	Procedures Needed	Logit Estimate [95% CI]	Outfit MSQ	Procedures Needed	Logit Estimate [95% CI]	Outfit MSQ
Endoscopy room setup	11–30	0.91 [95% CI: 0.65, 1.17]	1.53	11–30	−0.8 [95% CI: −1.0, −0.6]	0.78
Endoscopic equipment reprocessing	11–30	−0.16 [95% CI: −0.44, 0.13]	1.35	11–30	−0.74 [95% CI: −0.94, −0.54]	0.98
Post-procedure patient management	11–30	0.41 [95% CI: 0.14, 0.67]	1.41	11–30	−0.69 [95% CI: −0.89, −0.49]	1.15
Gastroscopy with biopsy	11–30	−0.55 [95% CI: −0.85, −0.25]	1.16	11–30	−0.63 [95% CI: −0.83, −0.43]	1.20
Colonoscopy with biopsy	11–30	−0.65 [95% CI: −0.97, −0.34]	1.18	11–30	−0.58 [95% CI: −0.78, −0.38]	0.71
Intra-procedural sedation management	11–30	−0.4 [95% CI: −0.71, −0.08]	1.13	11–30	−0.52 [95% CI: −0.72, −0.32]	1.12
Emergency endoscopy management	31–50	−0.38 [95% CI: −0.68, −0.08]	0.99	11–30	−0.47 [95% CI: −0.67, −0.27]	1.04
Injective endoscopic hemostasis	31–50	0.27 [95% CI: 0, 0.55]	0.82	11–30	−0.41 [95% CI: −0.61, −0.21]	1.21
Mechanical endoscopic hemostasis	31–50	0.12 [95% CI: −0.15, 0.39]	0.91	11–30	−0.36 [95% CI: −0.56, −0.16]	1.27
Thermal endoscopic hemostasis	31–50	0.14 [95% CI: −0.14, 0.42]	0.82	11–30	−0.3 [95% CI: −0.5, −0.1]	1.12
Topical endoscopic hemostasis	31–50	0.37 [95% CI: 0.09, 0.64]	0.76	11–30	−0.25 [95% CI: −0.45, −0.05]	0.94
ERCP: Stone removal, bile duct dilation, stent placement	11–30	−0.89 [95% CI: −1.62, −0.16]	0.95	11–30	−0.19 [95% CI: −0.39, 0.01]	1.27
Endoscopic ultrasound (with or without biopsy)	31–50	0.13 [95% CI: −0.2, 0.45]	0.93	11–30	−0.14 [95% CI: −0.34, 0.06]	1.18
Percutaneous Endoscopic Gastrostomy (PEG or PEJ)	31–50	0.1 [95% CI: −0.18, 0.39]	0.79	11–30	−0.08 [95% CI: −0.28, 0.12]	1.04
Ablation techniques (e.g., radiofrequency)	31–50	0.37 [95% CI: 0.03, 0.71]	1.08	11–30	−0.03 [95% CI: −0.23, 0.17]	1.42
Endoscopic Mucosal Resection (EMR)	31–50	−0.24 [95% CI: −0.59, 0.12]	0.65	31–50	0.03 [95% CI: −0.17, 0.23]	0.86
Endoscopic Submucosal Dissection (ESD)	31–50	−0.36 [95% CI: −0.72, 0]	0.72	31–50	0.08 [95% CI: −0.12, 0.28]	0.87
Endoscopic Full-Thickness Resection (EFTR)	31–50	−0.41 [95% CI: −0.79, −0.03]	0.86	31–50	0.14 [95% CI: −0.06, 0.34]	0.98
Zenker’s diverticulectomy	11–30	−0.18 [95% CI: −0.74, 0.36]	0.74	31–50	0.19 [95% CI: −0.01, 0.39]	1.43
Peroral Endoscopic Myotomy (POEM)	31–50	−0.12 [95% CI: −0.53, 0.29]	0.80	31–50	0.25 [95% CI: 0.05, 0.45]	0.96
Mechanical and pneumatic dilation	31–50	−0.1 [95% CI: −0.41, 0.21]	0.79	31–50	0.3 [95% CI: 0.1, 0.5]	0.74
Stent placement for strictures	31–50	−0.49 [95% CI: −0.82, −0.16]	0.68	31–50	0.36 [95% CI: 0.16, 0.56]	1.31
Enteroscopy	31–50	0.46 [95% CI: 0.14, 0.78]	0.75	31–50	0.41 [95% CI: 0.21, 0.61]	1.11
Capsule endoscopy	11–30	0.11 [95% CI: −0.2, 0.42]	0.96	31–50	0.47 [95% CI: 0.27, 0.67]	0.77
Esophageal manometry and pH monitoring	11–30	−0.26 [95% CI: −0.63, 0.11]	1.35	31–50	0.52 [95% CI: 0.32, 0.72]	1.30
Endoscopic management of inflammatory bowel disease	11–30	−0.77 [95% CI: −1.13, −0.41]	1.11	31–50	0.58 [95% CI: 0.38, 0.78]	1.03
Drainage of pancreatic or gallbladder collections	31–50	−0.11 [95% CI: −0.46, 0.24]	0.93	31–50	0.63 [95% CI: 0.43, 0.83]	1.48
Bariatric endoscopy	31–50	0.27 [95% CI: −0.08, 0.62]	1.09	31–50	0.69 [95% CI: 0.49, 0.89]	0.95
Fecal microbiota transplantation (FMT)	11–30	0.15 [95% CI: −0.35, 0.65]	1.36	31–50	0.74 [95% CI: 0.54, 0.94]	0.99
Nursing-led endoscopy research	31–50	0.25 [95% CI: −0.12, 0.62]	1.50	31–50	0.8 [95% CI: 0.6, 1.0]	0.73

Note: 95%CI = 95% Confidence Interval; MSQ = Outfit mean square. Legend: ERCP: Endoscopic Retrograde Cholangiopancreatography; EFTR: Endoscopic Full-Thickness Resection; EMR: Endoscopic Mucosal Resection; POEM: Peroral Endoscopic Myotomy; PEG: Percutaneous Endoscopic Gastrostomy; PEJ: Percutaneous Endoscopic Jejunostomy; FMT: Fecal Microbiota Transplantation; ESD: Endoscopic Submucosal Dissection.

## Data Availability

The datasets analyzed during the current study are available from the corresponding author upon reasonable request.

## Supplementary Materials

The following supporting information can be downloaded at: https://www.mdpi.com/article/10.3390/healthcare14020203/s1, Table S1, Strengthening the reporting of observational studies in epidemiology (STROBE) [25] checklist.
